# MAKING ANIMALS ALCOHOLIC: SHIFTING LABORATORY MODELS OF ADDICTION

**DOI:** 10.1002/jhbs.21715

**Published:** 2015-03-04

**Authors:** EDMUND RAMSDEN

## Abstract

The use of animals as experimental organisms has been critical to the development of addiction research from the nineteenth century. They have been used as a means of generating reliable data regarding the processes of addiction that was not available from the study of human subjects. Their use, however, has been far from straightforward. Through focusing on the study of alcoholism, where the nonhuman animal proved a most reluctant collaborator, this paper will analyze the ways in which scientists attempted to deal with its determined sobriety and account for their consistent failure to replicate the volitional consumption of ethanol to the point of physical dependency. In doing so, we will see how the animal model not only served as a means of interrogating a complex pathology, but also came to embody competing definitions of alcoholism as a disease process, and alternative visions for the very structure and purpose of a research field.

## Introduction

When reflecting on the use of animals in the medical sciences, biochemist David Lester considered them to have been critical to the study of reproductive physiology, cancer, gerontology, nutrition, albinism, and a whole range of disease processes from anemia to zoonosis, “the list … is long indeed, ranging from the cat to (probably) the zebu … but certainly including cattle, chickens, dogs, ducks, hamsters, horses, mink, mice, primates, rabbits, rats, sheep and swine” (Lester, 1982, p. 149). Lester had long been determined to add alcoholism to this list, his own research favoring the rat, the animal of choice in much of experimental psychology and physiology. This paper will examine the enduring attempt to make alcoholics out of animals in the laboratory, Anthony Riley and Cora Lee Wetherington (1989, p. 205) asserting: “In few places has [the] effort to establish an animal model been as extensive as in the specific pathology of alcoholism.” It will not restrict itself to the issue of choice of animal, as important as this is, but rather focus instead on the various techniques and methods used in order to realize an animal model of alcoholism.

In doing so, this paper will contribute to our understanding of the history of the science of alcohol use and abuse, a history that has been little explored, historians preferring to focus on the social and political debates surrounding the control of alcohol consumption. Due to the perceived complexity of alcoholism as a disease, differences of opinion as to etiology, treatment, and prevention, and ethical restrictions regarding human experimentation, the animal laboratory has been a particularly important site for the study of the phenomenon. The importance of animals in addiction research more generally, particularly that involving opiates and cocaine, has been subject to greater historical attention. However, in these studies, the animals used served as passive and reliable producers of data, the degree of control and replicability that they provided obscuring the complexities of human drug use and abuse. In her study of the search for a nonaddictive analgesic, Caroline Acker describes how pharmacologists treated the laboratory animal as a “black box, receiving the input dose and exhibiting a measurable response” (1997, p. 144). A “tail flick test” became the standard measure for analgesic potency, obstructing the search for drugs with therapeutic potential.^1^ In her account of the creation of “junkie monkeys” in addiction studies, Nancy Campbell (2007) describes how pharmacologists effectively bracketed “desire” through a “laboratory logic” that helped limit attention to interaction between organism and drug. They skirted difficult issues, such as the effects of social dynamics, complex environmental and psychosocial conditions, subjective cravings and internal states by defining addiction in terms of the physical reinforcing effects of the drug, measured in animals through increased tolerance, dependence, and symptoms of withdrawal.

For those seeking to understand the causes of alcoholism, however, the creation of an animal model was particularly complicated. While an animal might choose to drink alcohol, it did not do so in significant amounts or for its intoxicating qualities. The “effort” expended in creating an animal model was, therefore, a measure of the difficulty of realization. For this reason, it is particularly useful for examining the practices of animal modeling. As Rachel Ankeny (2010) argues, historians have been constrained by a near exclusive attention to particular kinds of modeling. The historical emphasis has been on genetics and genomics, resulting in a focus on a relatively limited number of species that can be easily analyzed using genetic techniques, on molecular‐level processes, simplification, and the methods of standardization. Ankeny calls on historians to broaden their perspective, looking beyond the ubiquitous “model organisms” of genetics, where the animal has become standardized and packaged, its purpose accepted, and its use near universal. In order to understand the emergence of model organisms, more attention needs to be focused on the wide range of “animal models” constructed and used in a variety of disciplines and research programs, including the “outliers,” “failures,” and “losers” in animal modeling and experimentation (Ankeny, 2010, p. 100).

In recent years there has been much greater attention given to the difficulties, complexities, and general messiness of animal modeling. As one of the subjects of this essay, the behavioral psychologist John Falk observed with regards operant conditioning, a paradigm so often seen as synonymous with the ideals of objectivity, predictability, and control: “The course of true reinforcers seldom runs smooth” (1983, p. 389). Modeling is a complicated and unpredictable business, particularly so when the animal is relied upon to act and behave in a way that has value to the experimenter. Yet, as Rheinberger (1997) argues, deviations from the expected are critical to the success of any experimental system, while, for Wimsatt (2007), models often misrepresent or fail to predict, but even when “false,” they can prove particularly effective tools for developing “truer theories.” Morrison and Morgan describe models as tools that mediate between the domain of “things” and the domain of “theory”; they have a “life of their own,” and while they operate on a scientist's behalf, they serve as useful instruments of learning precisely because they retain a degree of autonomy (Morrison & Morgan, 1999, p. 18; cf. Ankeny & Leonelli, 2011). Laboratory animals, part artifact, part “sample of nature,” are particularly adept at generating new scientific problems and questions (Rheinberger, 1997; Leonelli, 2007). The success of an experimental system therefore depends upon the abilities of scientists to reframe continuously their theoretical outlines and agendas in conjunction with varying and mutable laboratory materials (cf. Griesemer, 1992). As scholars have argued, the “rightness” of a tool for a job is not predetermined by, say, the specific biological qualities of an organism, but “*co‐constructed*, mutually articulated through interactions among all the elements in the situation” (Clarke & Fujimura, 1992, p. 5).

This paper will focus on the problems that emerged when scientists attempted to build a model of a disorder, the different ways in which they attempted to deal with these problems, and, finally, how they made these problems work to their advantage in terms of promoting their own visions of the ideal structure, methods, and contributions of a scientific field. The difficulties in constructing an animal model of alcoholism generated many questions regarding the relationship between modeling and the disease or disorder it was to represent: What should an animal model of alcoholism look like? What characteristics should it contain? What criteria should it meet? Should there be one model or many models? What uses should it, or they, serve? As we shall see, the criteria developed to define the parameters of an animal model of alcoholism served a dual purpose. They were a means of delineating and understanding the phenomenon in question, and a means of circumscribing and organizing a scientific field and therapeutic approach—what belonged inside and what lay outside the model, and how certain characteristics were aligned in relation to one another, reflected conceptions of how the alcohol research field should be structured and alcoholism treated.^2^ Focusing on a research field in which there is both considerable dedication to, and difficulty in, creating a model of a phenomenon—the excessive and compulsive drinking of alcohol to the point of physical dependency—brings this mediating function into sharp relief.

This paper will pay particular attention to the debate over a proposed solution to the determined sobriety of experimental animals—that of schedule‐induced polydipsia (SIP)—a surprising and unexpected by‐product of operant conditioning. This approach was very successful in inducing animals to drink alcohol compulsively and to excess, but not in generating sustained drinking outside of the experimental situation. We therefore have an opportunity of exploring how different communities of scientists interpreted and explained the apparent discrepancies and lack of fit between representation and phenomenon. In deeming the approach a failure, scientists sought to develop new and stricter standards for modeling that demanded the replication of the disease of alcoholism in the human in its essential characteristics. This would not only improve methods of medical intervention, but also help secure the scientific status of the alcohol research field. However, others reinterpreted the very failure to represent alcoholism as a disease as a success, demanding a reinterpretation of the phenomenon being modeled, and a fundamental reorganization of a scientific field of study and approach to therapy.^3^ The unpredictable, active, and often intractable problems involved in modeling are put to work not only in the context of scientific discovery, but also in the justification of an experimental system and in the construction and delineation of a scientific field. This paper will, therefore, not only demonstrate the significant role of animal modeling to the development of the alcohol research field, but that this importance did not necessarily depend upon the successful replication of the phenomenon being modeled, but rather, on the effective interpretation, framing, and application of its presumed shortcomings.

### The New Approach to Alcoholism

Following the repeal of prohibition in 1933, there began what the biometrician and physiologist E. M. Jellinek described as a “new approach to alcoholism.”^4^ Previously, he argued, alcohol abuse was primarily a legislative, moral, and ethical issue; it was now a medical problem and a subject of wide‐ranging scientific study. In 1937, the National Academy of Sciences established a Research Council on Problems of Alcohol, and from 1940 to 1950, it supported a Section of Studies on Alcohol at the Laboratory of Applied Physiology at Yale. This became the Center of Alcohol Studies (CAS) in 1950—the focal point of alcohol research and publisher of the *Quarterly Journal of Studies on Alcohol*. In 1940, Howard Haggard, the Director of the Laboratory of Applied Physiology, invited Jellinek to Yale, and here he rapidly became the world's leading expert on alcohol problems. For Jellinek, alcoholism was a disease; it was not a moral failing, or the inevitable consequence of prolonged drinking, and neither was it simply a symptom of an underlying mental illness, treatable through psychotherapy or psychoanalysis.^5^ Jellinek's approach proved popular because it provided coherence, clarity, and, above all, it removed stigma—alcoholism was not due to weakness of will or temperament, but was a serious physical illness involving a pharmacologically addictive substance that afflicted members from all sections of society.^6^ From 1951, Jellinek served as a consultant to the World Health Organization, ensuring that the Alcoholism Sub‐Committee of the Expert Committee on Mental Health extended its purview beyond psychiatry to address pharmacology and physiology. The World Health Organization (WHO) 1954 declaration that alcoholism was a disease and an immense public health problem reflected his considerable influence (WHO, 1955). This was followed by a resolution in 1956 by the American Medical Association that Jellinek (1960, p. 164) declared as the “formal acceptance of the disease conception of ‘alcoholism’ by the American medical profession as a whole.”

The understanding and promotion of the disease conception was dependent upon scientific research—the primary function of the CAS. For Jellinek, the study of alcoholism was fundamentally multidisciplinary, and the CAS employed biochemists, sociologists, anthropologists, physiologists, and psychologists. Within the CAS structure, basic, laboratory‐based research was preeminent.^7^ Jellinek considered empirical verification of some the physical effects of alcohol on physiological functions as well established, stimulated by earlier debates over temperance, and much of it involving experiments with animals.^8^ This work did not, however, “explain *why* the person had been drinking to excess for years; any theory of the etiology of addiction must answer this question, i.e., must show the driving forces, psychological or physiological, which cause this drinking” (Bowman & Jellinek, 1942, p. 14). When it came to the “motivation in the genesis of the alcohol habit,” psychiatric and psychological speculation was rife and experiment offered a complete “*tabula rasa*” (Jellinek & McFarland, 1940, p. 276). Animal research was critical to this new experimental phase, as it allowed for the analysis of the psychophysiological mechanisms involved in the development of the disease that would not have been possible with human subjects.

Particularly important to the developing field of alcohol research was the emergence of an experimental psychiatry. W. Horsley Gantt at Johns Hopkins, and Jules Masserman, first at the University of Chicago and then at Northwestern, were leaders in this field, which rose to a prominent position in the 1940s. It was by turning to the controlled environment of the laboratory that the various environmental and biological factors underlying mental disturbances could be identified and understood, helping to realize the more holistic and dynamic psychobiological psychiatry promoted by Adolf Meyer.^9^ As Masserman argued, the behavior of nonhuman animals was intelligent, willful, purposive, dynamic, and motivated by “goal‐directed strivings” (1943, pp. 6, 7, 9), differing from the human only in terms of their “technics of adaptation.” Consequently, nonhuman animals also suffered from motivational conflicts and emotional disorders. Gantt and Masserman further developed Pavlovian techniques for producing “experimental neurosis,” involving the pairing of competing drives of excitation and inhibition, resulting in a range of striking behaviors—extreme agitation, muscular tension, accelerated pulse and raised blood pressure, asthmatic breathing, gastrointestinal disturbances, inertia, hostility, phobias, and even a range of sexual deviations.

Such an approach not only provided an understanding of the generation of mental disturbances, but the effects of various factors on those disturbances once established. These included drugs such as alcohol. In the late 1930s, Gantt plied one of his experimental animals with alcohol, a dog with “anxiety‐like neurosis of long standing,” and found its “frequent pathological erections and ejaculatio praecox” corrected (1952, p. 180; cf. Gantt, 1940). Masserman carried out extensive studies using neurotic cats. Having learned a complex response (opening a box when signaled) in order to obtain food, and subsequently receiving an electric shock or air blast when doing so, the cats were able to overcome the resulting impasse between hunger and fear when intoxicated (Masserman et al., 1944; Masserman, Jacques, & Nicholson, 1945; Masserman & Yum, 1946; Masserman, 1957). Masserman postulated that this “antineurotic” effect (1957, p. 164), could be explained by the ability of alcohol to “disorganize the complexly aberrant patterns of recently‐induced experimental neuroses and permit a reversion to the simple, more directly goal‐oriented responses of more relatively ‘normal’ behavior” (Masserman & Yum, 1946, pp. 48–49). Having established its ability to mitigate neurotic behavior, Masserman then explored the power of alcohol to “prevent the consolidation of perceptive, integrative and reactive processes during the conflictful experience and so prevent the development of neurotic reactions” (Masserman, Jacques, & Nicholson, 1945, p. 281). Once again, when inebriated, the animal's reactions to stress were milder and there was no persistent phobia or anxiety.

CAS researchers built upon Masserman's studies, identifying how alcohol diminished intense “emotional response,” involving manic behavior, convulsions, and catatonia‐like immobility (Greenberg & Lester, 1953; cf. Dember, Ellen, & Kristofferson, 1953). However, they also began to turn towards psychology for more simplified methods of producing conflict and for more straightforward and general conceptualizations of learned patterns of anxiety and fear. Most important in this regard was the work of John Conger, a psychologist who had trained under the behaviorists Neal Miller and John Dollard at Yale. Conger argued that the reinforcement theory of learning obviated Masserman's highly complex formulations. What was at play was simply a learned fear response, conflicting with the drive for food, that the animal was able to overcome through the tension relieving effects of alcohol. He devised a series of experiments involving an “approach‐avoidance conflict” situation. Rats, once trained to approach the lighted end of a straight‐line alley to secure food, received an electric shock at the goal. Once injected with alcohol, they were able to overcome the resultant conflict, again entering the alley for the food reward in spite of the possibility of electric shock. Drinking was learned because it was rewarding—alcohol served to reduce fear, and thus, the reinforcing qualities of alcohol were to be understood in relation to its “social‐emotional effects” (Conger, 1951, p. 24).

Masserman and Conger both interpreted their findings as directly supporting Jellinek's belief in the tension, reducing effects of alcohol and its value as a source of “emotional relaxation” (Greenberg & Lester, 1953, p. 389). Conger (1958, p. 36) emphasized that it was through the study of animals that they had finally been able to address such a complex topic as the motivational basis for continued drinking, providing “support for the clinical observation that alcohol reduces the tension resulting from fear or anxiety, thus helping to restore a state of equilibrium in the individual.” Masserman described the stresses in his animals as “at least partly analogous to conflict‐engendering situations in humans” (1957, p. 147). Through his study of cats in a cage, the value of alcohol was clear: “men long ago learned to drink alcoholic beverages either as a ‘bracer’ to cloud and thereby mitigate the anticipated stresses of impending experiences, or as a hypnotic that blunts and disorganizes neurotic anxieties and symbolic hypersensitivities” (Masserman, Jacques, & Nicholson, 1945, p. 298).

Throughout the 1940s and 1950s, there were numerous other animal studies that associated the reinforcing properties of alcohol with its ability to reduce fear and anxiety.^10^ Tension and drive reduction exemplified American psychology during this period, as Greeley and Oei (1999, p. 14) have argued, providing the impetus and motivation for behavior and the underlying mechanism of reinforcement. However, in the majority of experiments the animals were either force‐fed or injected with alcohol. Gantt described having to “coax” his dogs to ingest an alcohol‐milk solution, often resorting to gastric intubation. Masserman had more success. He noted that “normal cats” ordinarily refuse alcohol, so he deprived them of fluids for up to 96 hours, and even then, a stomach tube was commonly required (Masserman, Jacques, & Nicholson, 1945, p. 283). Having repeatedly experienced relief from neurotic tensions through alcohol, he claimed that approximately half of his cats developed a preference for “Alexander cocktails of milk spiked with alcohol” over plain milk (Masserman, 1957, p. 159). Nevertheless, not only was this preference short‐lived, and “only at the limen of statistical reliability” (Masserman & Yum, 1946, p. 50), but, as psychologists noted, it also took place in the same stimulus complex in which conflict had been generated and there were no measures of the amount consumed (Korman & Stephens, 1960). Other researchers struggled to replicate Masserman's limited success, the overwhelming conclusion being that, even with its apparent tension relieving properties, few animals preferred alcohol solutions, and thus, few considered it significantly rewarding.

Others turned to another possibility for understanding the genesis of the alcohol habit that had emerged out of earlier psychophysiological studies. The psychobiologist, Curt Richter, was, like Gantt, employed at Meyer's Phipps Psychiatric Clinic, and shared the interest of his colleagues in the homeostatic mechanisms of mind and body. However, for Richter, physiology determined behavior. In his work on spontaneous activity and self‐regulatory functions, he had allowed his experimental animals—the Norway rat—to select its own diet, believing that the “wisdom of the body” ensured that an animal chose the correct nutritional requirements. Adrenalectomized rats survived by ingesting large amounts of salt, while parathyroidectomized rats duly selected solutions with large amounts of calcium (Richter, 1941a). Richter believed that the rat was the ideal animal for understanding human psychophysiology, and described their dietary requirements as “almost identical” (Richter, 1943, p. 94). This extended to alcohol, Richter discovering that his animals preferred a solution of approximately 5 percent in concentration, and chose wine and beer in a free‐choice situation, when paired with plain water (Richter & Campbell, 1940; Richter, 1941b). This was a significant development when we consider how others had relied on needles, force‐feeding tubes, and gastric fistula.

Richter's work was very influential, reinvigorating a physiological approach to alcohol problems through the animal laboratory. This “experimental alcoholism” no longer merely focused on the pathological effects of alcohol on the body, but on how preexisting physical pathologies generated addiction (Mardones, 1951, p. 563). Applying the logic of homeostasis, Richter focused on the physiological causes of variations in selections of alcohol among his animals. He suggested that perhaps the “spree” drinking that characterized alcoholics resulted from thyroid deficiencies, contributed to by the high‐calorie content of alcohol (Richter, 1957, pp. 117, 124, 125). Others focused on specific vitamin deficiencies, most notably that of thiamine. Jorge Mardones, a biochemist and pharmacologist from the University of Chile, described having been “deeply impressed” by Richter's work (Mardones, 1991, p. 386). In 1942, he began by feeding rats a diet deprived of thiamine (autoclaved yeast), and found that the self‐selection of alcohol increased. When given a supplement of untreated dried yeast or liver, their alcohol intake duly declined (Mardones, 1951). Roger J. Williams, a biochemist and nutritional specialist at the University of Texas at Austin, similarly confirmed that rats with diets deficient in vitamin B complex increased alcohol intake under conditions of self‐selection (Williams, Berry, & Beerstecher, 1949). For Williams it seemed clear that alcoholism was not the result of a psychological craving, but “a deranged *physiological craving induced by a physiological agent*” (Williams, 1959a, p. 17). He suggested that it was an example of a “genetotrophic disease”: an individual with an unusually high nutritional requirement of a genetic origin would select alcohol due to its value as a food. However, the alcohol would then interfere with the individual's appetite controlling centers and nutritional balance, leading to the selection of yet more alcohol, and so on, as “body wisdom changes to body foolishness” (Williams, 1959a, p. 48). In order to identify those mechanisms that determined alcohol preference, both Mardones and Williams began to breed “drinker” strains of rats that chose alcohol in Richter's free‐choice situation (Mardones, Segovia, & Hederra, 1953; Williams, 1956).

In this new physiological approach to alcoholism, the nutritive value of alcohol was the key to drinking, Mardones describing alcohol as “at the same time a nutrient and a drug” (Mardones, 1991, p. 386). However, for critics of such as “deceptively simple” interpretation, it also lent itself “to simple, and artful, proofs” (Lester & Greenberg, 1952a, p. 450). CAS researchers found that when offered a “third” choice—that of a sugar solution—the alcohol intake of the animals dropped dramatically. This suggested that the animals were using alcohol simply as a means of supplementing the lack of calories in the unsatisfying “mush” served in the laboratory (Greenberg & Lester, 1952a, p. 451). While Mardones and Williams may have sought to overcome criticism with drinker strains, this led to a second closely related problem: the animals were not drinking to get drunk, and thus, for the pharmacological properties of alcohol. Richter admitted: “I have never seen an intoxicated rat” (1957, p. 81), and Mardones: “we have never observed rats with symptoms of intoxication” (1955, p. 53). Therefore, Lester and Greenberg (1952b, p. 559) argued, the extension of the genetotrophic argument to human alcohol was unwarranted “because the behavior of the animals in these experiments does not parallel the behavior of the human alcoholic. Alcoholics seek intoxication. The rats, though offered the opportunity to drink alcohol freely, never became intoxicated.” Once again, researchers were coming up against a seemingly intractable problem—the determined sobriety of their experimental animals.

In his field‐defining book of 1960, *The Disease Concept of Alcoholism*, Jellinek reviewed the experimental evidence provided through animal laboratories. Jellinek was attempting to mold multifarious theories and data into a coherent “representative model” of alcoholism as a disease. Seeking clarity through definition, he distinguished between various stages or “species” of alcoholism. “Alpha” alcoholism was the early stage of alcoholism, where alcohol was used to alleviate psychological stress and emotional pain; “beta” alcoholics were heavy drinkers, but were not psychologically dependent; “gamma” alcoholics exhibited regular bouts of extreme uncontrolled drinking; “delta” alcoholics were more measured in their drinking, but were unable to abstain.^11^ Of these species, only two were diseased—gamma and delta—due to their respective characteristics of “loss of control” and “inability to abstain.” For Jellinek, the key feature of the disease process was the overwhelming desire for drink, overriding an individual's will: “the act which results in intoxication is outside the volitional sphere of the alcoholic” (Jellinek, 1960, p. 45). While the desire for alcohol in the early stages of alcoholism had important psychological components, proof of this disease process was to be found in physiology, “since it is the adaptation of cell metabolism, and acquired tissue tolerance and the withdrawal symptoms, which bring about ‘craving’ and loss of control or inability to abstain” (Jellinek, 1960, p. 40). The reality of alcoholism as a disease was observable and measurable through increased tolerance and withdrawal, and thus, was increasingly associated with these physical symptoms.

Jellinek then compared the results of the existing animal research to the characteristics of alcoholism as outlined in his representative model. He stressed the importance of Masserman and Conger's experiments for having identified alcohol as a depressant, rather than a stimulant, and for ascertaining its tension and anxiety‐relieving properties, giving it value to the would‐be alcoholic.^12^ He also acknowledged the work pioneered by Richter, Williams, and Mardones (dedicating the volume to the latter, “in friendship”) for identifying the importance of hereditary factors in alcohol preference. Where both these approaches fell short, however, was in their failure to generate the symptoms of addiction in the laboratory. He took issue with the psychologists’ assumptions that they had shown alcoholism to be a “learned addiction”:

*The criteria of addiction, in the proper pharmacological sense, are not fulfilled in animal experiments. Animals to not reach a stage of senseless intoxication, they do not exceed the aim of alcohol intake by quantities which bring about a cancelation of the adaptive functions of alcohol*. (Jellinek, 1960, p. 76)

Similarly, with regards the “experimental alcoholism” of Williams and Mardones, a preference for alcohol was not a “craving”: “It may be noted that rats which increased their voluntary intake of alcohol did not exhibit those phenomena that are associated with the picture of alcoholism” (Jellinek, 1960, p. 97). He was also critical of the tendency of both sets of researchers to promote their approach at the expense of the other—that alcoholism was purely a question of learning, leading to habituation, or that a person was a genetically determined alcoholic before even touching a drink, both of which deemphasized the specific pharmacological qualities of alcohol. Seeking to counter one‐dimensional and oppositional stances, Jellinek criticized researchers for overextending the evidence from animal laboratories.^13^ He restricted the relevance of existing animal studies to “understanding the process that precedes the addictive phase of drinking,” such as in the stage of alpha alcoholism (Jellinek, 1960, p. 76). There was, thus far, no evidence of a progressive and overpowering compulsion to drink among animals, leading to increased tolerance and withdrawal, and thus, no evidence of a disease process.

### Schedule‐Induced Polydipsia

At the beginning of the 1960s, David Lester of the CAS reflected on the state of animal research in the alcohol field. Even an alcohol‐drinking rat, presented with alcohol as the sole fluid, would not become intoxicated; it seemed truly “impossible to attain and maintain the alcohol intoxicated state in experimental animals” (Lester, 1961, p. 223). However, he also expressed excitement at the potential of a new discovery. In 1961, the Canadian behavioral psychologist John L. Falk published a paper in which he described a “dramatic,” “unexpected,” and “curious” behavior among his laboratory rats (Charles River albino; Falk, 1961, 1971, 1981). Trained at McGill, followed by doctorate in experimental psychology at the University of Illinois Urbana, Falk was now a postdoctoral fellow at the Department of Nutrition, Harvard University School of Public Health. He described having had “complete research freedom” to combine his longstanding interest in physiological psychology with his growing fascination with the techniques of operant conditioning that he had learned from Charles Ferster and Roger Kelleher when employed for a short period at the Yerkes Primate Laboratory (Falk, 1987, p. 128; Dewsbury, 2003; Schuster, 2010). He was interested in seeing if rats would increase their lever‐pressing behavior when given bilateral, ventromedial hypothalamic lesions. He placed the rats on an intermittent variable interval one‐minute food schedule, in which a lever press delivered a small (45mg) pellet intermittently, from a few seconds to 2 minutes. He was also interested to see the effect on the relation between food‐pellet ingestion and water intake, and placed a water bottle in the cage. Before he even had a chance to inflict the lesions, he noticed that the rats drank after the delivery of every pellet, consuming half their own body‐weight in water in three hours. Falk's postgraduate training had been in the field of thirst, and he recognized that this intake was “strange and unprecedented” (Falk, 1969, p. 569; cf. Falk, 1987), to the point of being “sure that the drinkometer spout was leaking” (Falk, 1964, p. 97). Falk named his discovery *schedule‐induced polydipsia* (SIP), polydipsia meaning “excessive thirst.”

Falk's discovery attracted little interest in water balance circles. Researchers were committed theoretically to the idea of physiological homeostasis, contributed, in no small part, by the work of Richter. They deemed excess in self‐administration without a direct physiological modification of the animal, an anathema (Falk, 1987, p. 128). Falk questioned dipsologists inference of a physiologically determined motivational state: “we can no longer depend upon a correlation between the response and some internal regulatory mechanism.” In the place of a “thirst drive” or “regulatory adjustment,” environmental factors, in this case, “certain schedules of reinforcement are among the most potent known thirst inducing agents” (Falk, 1969, p. 586). However, as important as operant conditioning was to Falk's methodology, polydipsia also did not make behavioral sense—if an animal's behavior was determined by its consequences, that it was rewarding in some way, this behavior seemed “absurd”:

It was absurd because food deprivation in rats yields a decrease in water intake, not an increase. It was absurd because heating a large quantity of room‐temperature water to body heat and expelling it as copious urine is wasteful for an animal already pressed for energy stores. It is absurd for an animal to drink itself into a dilutional hyponatremia bordering on water intoxication. But perhaps most absurd was … the lack of an acceptable behavioral account. (Falk, 1971, p. 577)

He rejected behaviorists’ suggestion that such “schedule effects” could simply be explained through Skinner's concept of “adventitious reinforcement” (Clark, 1962; Stein, 1964; Segal, 1965). Skinner (1948) had observed the development of a “ritualistic” stereotyped pattern of behavior among pigeons during the interval of a 15 second fixed schedule—birds walked in circles before food presentations, scratched the floor, or moved their heads in a pendulum motion. Seeking to explain such seemingly unexpected and uncontrolled effects through the framework of operant conditioning, Skinner argued that the animals had come to associate a random action just prior to the appearance of food with its presentation. Thus, he was able to interpret the animal's behavior as determined by the reward, resulting in the accidental reinforcement of “superstitious” behavior. This theory of superstition seemed incompatible, however, as the animals drank immediately after the delivery of the food pellet, not before (Falk, 1969, pp. 574–575). Seeking an alternative explanation, Falk turned to the ethology of Niko Tinbergen (1952). Behaviors that seemed misplaced or “out‐of‐context’’ in relation to the environment by not serving an appropriate motive or activated instinct were “displacement activities”—birds suddenly ceasing to fight to engage in preening, for example. When an individual animal was unable to do something that they were motivated to do, they would turn to an easy and satisfying alternative—an “adjunctive behavior”—allowing for the stabilization of irreconcilable vectors. It was an alternative to the extreme, and potentially disastrous, choices of fight or flight. Falk's rats were hungry and wanted to eat. Unable to perform this desired behavior, they did whatever else was available to them: drinking. He noted how SIP could be induced using a variety of different liquid solutions, and in different species (Schuster & Woods, 1966; Falk, 1969; cf. Meisch, 1969; Shanab & Peterson, 1969).

In contrast to dipsologists, alcohol researchers were quick to identify the “first‐rate importance” of Falk's discovery (Lester, 1961, p. 224). They were little interested in the broader theoretical implications of adjunctive behavior. What they were interested in was the technique. Lester replaced the water used in Falk's experiments with a 5.6 percent alcohol solution, and managed to maintain “a state of inebriation or near inebriation during some 65 hr.; at no time did the animal become sober.” Continuous measurement of blood alcohol levels throughout the experiment even suggested “the development of a metabolic tolerance” (Lester, 1961, p. 227). The method was also attractive because of the precise measurements that it provided—each lick and bar‐press recorded and quantified. In the 1960s, addiction researchers more generally were drawing on the techniques of behaviorism, while Skinner, in turn, promoted the relevance of his methods to pharmacology (Skinner & Heron, 1937; Ferster & Skinner, 1957; Skinner, 1959; cf. Thompson & Schuster, 1968; Pickens, 1977). The technologies of operant conditioning provided a highly stable baseline of behavior against which the effects of drugs could be measured with precision over time. They also provided metric criteria of habituation in the place of the more obscure and subjective notion of “craving” (Casey, 1960, p. 208). Researchers could establish the reinforcing properties of a solution and quantify the preferences of animals; the number of lever presses as a measure of how hard an animal would “work” to receive a substance became a central feature of the “behavioral criteria of addiction” (Mendelson & Mello, 1964, p 3; cf. Myers, 1961; Mello & Mendelson 1964). The experimenter could also induce an animal to take various drugs as secondary reinforcers—hungry animals consuming alcohol for a reward of milk, or imbibing to avoid electric shock (Mello & Mendelson, 1965; Persensky, Senter, & Jones, 1968; Senter & Persensky, 1968; Keehn, 1969). Yet with polydipsia, there were no such contingencies necessary or apparent, and psychologists proficient in operant conditioning quickly took to Falk's method (Holman & Myers, 1968; Mello & Mendelson, 1971a; Meisch & Thompson, 1972; Meisch, Henningfield, & Thompson, 1975).

Lester believed that the major obstacle in alcohol research had been overcome by a “simple means”: not only did the animals drink large amounts, but “the drinking, moreover, had the appearance of being compulsive in character” (Lester, 1961, p. 224). In a CAS report of 1966, Lester stated that while all previous attempts to increase alcohol consumption in the rat had failed, “it is fortunate that at least one technique now makes it possible to determine the factors responsible for the acceptance or rejection of large amounts of alcohol by an infra‐human organism.”^14^ By further modulating the level of ingestion by adjusting the experimental parameters (operant schedules, liquid choices) controlling this behavior, there seemed potential for “illuminating dark corners in the irrational behavior of man, and specifically the addiction to alcohol.”^15^ However, this meant that alcohol researchers were faced with another problem—explaining the phenomenon in a way that seemed relevant to the issue of alcoholism. Seeking to interpret the behavior in the context of a well‐established paradigm of alcohol research, Lester speculated that perhaps the “unpredictable occurrence of a food reward is an anxiety‐producing stress in the rat,” and that by further modifying the program “so as to increase the magnitude of this factor will also increase the drive to drink” (Lester, 1961, p. 230). The ways in which different communities of researchers interpreted the process, relevance, and value of SIP reveals fundamental differences regarding the role of the animal model, the structure of the alcohol research field, and the very definition of alcoholism as a disease.

### Problematizing SIP and Establishing Standards: The True, the Total and the Partial

CAS researchers had been the first to promote the relevance of SIP to the study of alcoholism. Yet, they were also the first to question its value. Just as in their assessment of the alcohol preference paradigm, CAS researchers sought to differentiate between the influence of alcohol's nutritional and pharmacological properties. The standard practice in behaviorist research was to give the reinforcer more value to the animal, and Falk had duly reduced his rats to 80 percent of their free‐feeding weight. Many psychologists were criticizing such “partial starvation” for introducing significant experimental bias (cf. Dewsbury, 2003), a problem that was even more relevant in the case of alcohol. David Lester carried out a series of studies with Earl Freed, suggesting that “caloric need” was the cause of polydipsic drinking (Freed & Lester, 1970; Freed, 1972). The extrapolation from polydipsic animals encountered the same problems faced by Richter's alcohol preferring rats: “few, if any, human alcoholics become alcohol‐dependent because the beverage tastes or smells so delectably or becomes more appealing than other foods” (Myers & Veale, 1972, p. 147).

The second problem was that of dependency. Researchers found that although they were able to increase the alcohol drinking of animals through SIP, when they returned them to their home cages, there was a rapid extinction of excessive drinking and their alcohol preferences returned to the normal range (Senter & Sinclair, 1967; Mello & Mendelson, 1971a). They were not addicted, and it seemed that compulsive drinking was purely a figment of the experimental situation. This led to the thorny issues of volition and motivation. Polydipsia was the consequence of a process that they did not yet fully understand; it therefore “seems hardly possible for the authors to state flatly that the drinking is not dependent on any contingencies” (Friedman & Lester, 1977, p. 5). SIP seemed an anomaly, an aberrant response dictated by the specific, isolated, and artificial experimental environment imposed by operant conditioning. While “impressive… at first glance,” it was “but a mimicry of man's abuse of alcohol,” a mere imitation through a “surface” equivalence; they were not modeling a functional relationship: “where the alcoholic drinks from strong inner motivations, the rat is constrained to do so by equally compelling external manipulations alien to man” (Lester & Freed, 1973, p. 105). SIP was “not a model,” as the basis of the animal's excess was not the “same” as in man, where it was due to the “effects of alcohol as discomfort attenuation, analgesic, tranquilizing, and palliative action, and as an intoxicant with euphoria‐producing characteristics” (Lester & Freed, 1972, p. 58).

In the language used by Lester and Freed, we can see a determination to establish clearly the standards required of an “animal model” of alcoholism. They listed the criteria that would have to be met: the animal orally ingesting substantial amounts of alcohol without food deprivation and with competing fluids available; ingestion be directed to the central intoxicating character of alcohol; the animal working to obtain it, even overcoming obstacles to do so; intoxication be sustained over a long period resulting in withdrawal syndrome and physical dependence; after abstinence, the animal reacquire its preference for intoxication (Lester & Freed, 1973, p. 106). Thus, the animal not only had to drink alcohol in pharmacologically significant amounts, but it also had to do so in a meaningful way—that is, for the same reasons as man. They reflected on the purpose of a model:

As conventionally conceived an animal model is a *miniature representation, perhaps altered in scope and scale, but not in its essence, which is brought into the laboratory. Accepting this, the researcher's task becomes clear: to be sufficiently conversant with the dimensions and parameters of the original so that they can be appropriately duplicated, in this case not necessarily by reduction as much by transference to another organism*. (Lester & Freed, 1973, pp. 103, 104)

The “dimensions” and “parameters,” as identified in Jellinek's conceptualization of a disease process, included a motivation to drink driven by alcohol's “anxiety‐attenuating influence,” leading to reinforcement, and then to increased tolerance, physical dependence, and withdrawal symptoms. For an animal model to be realized, these features needed to be represented in “a one‐to‐one relation” (Lester & Freed, 1973, p. 103). They were dismissive of the idea that SIP served as an animal model of alcoholism:

*While not advocating perfection, we do recommend that the phrases “animal model” and “addiction model” be parsimoniously reserved for animal behavior which stringently meets both psychological and physiological criteria*. (Lester & Freed, 1973, p. 106)

Others developed similar criteria. The psychologist T. J. Cicero's influential description of a “true model of alcoholism” encompassed “all the key features of the human condition”: the oral self‐administration of ethanol in a pharmacologically significant amount in the presence of other liquids; consumption solely due to its pharmacological properties; continuous consumption, leading to tolerance and physical dependence (Cicero, 1979, pp. 534, 535). At present, there was no single analogue that met all these criteria; thus, “in a strict sense there is no animal analogue of alcoholism” (Cicero 1979, p. 535). However, Cicero and other researchers also wished to shore up the evidence and advantages obtained through animal research. They made it clear that this “total” animal model was an “ideal”; the failure to realize the “true” analogue did not diminish the value of existing experimental preparations. If a researcher was looking at the effects of alcohol on the body, for example, the criteria of volition was irrelevant. Excluding complex “psychosocial variables,” if one then “dissects human alcoholism into its separate components, there may in fact be animal models” (Cicero, 1980, p. 100):

We do not have a complete animal model of alcoholism, but we may have models to examine the excessive intake of alcohol, and we certainly have models which permit an examination of the development of tolerance, withdrawal behavior, and the biomedical complications associated with alcoholism. (Cicero, 1980, p. 101)

Other researchers emphasized the value of “partial” analogues still further. Pursuing a behavioral genetic approach, David Rodgers (1966, p. 499) divided alcoholism into a series of “behavior segments,” reflecting the progressive phases identified by Jellinek: “In our studies of mice, we have therefore concentrated primarily on the parameter of voluntary alcohol consumption, because we think this is the appropriate segment at the animal level for understanding the pathology of alcoholism.” His one‐time colleague, Gerald McClearn, also developing alcohol‐preferring mouse strains (Rodgers & McClearn, 1962), freely confessed that his own work had failed to meet Lester and Freed's (1973) criteria: “However, while awaiting the development of such a model, I think our time can be well spent in research that utilizes partial models” (McClearn, 1979, p. 255). McClearn (1979, p. 256) favored the term “simulacrum” that, as “a representation of something,” had a “ring of modesty about it that seems appropriate for the limited‐scope models that have been employed.”

In spite of the differences in emphasis between these researchers, they shared common concerns and commitments. The true or total animal model of alcoholism eluded them, but it remained an important and unifying ideal for them to work toward and realize in time. Rodgers spoke of the necessity of an “integrated approach” that subjected various stabilized strains to differing environmental manipulations and stimulus conditions, such as stressful situations (1966, p. 500). Cicero, and his colleague and mentor Robert D. Myers, were particularly active in combining genetic, behavioral, biochemical, and physiological approaches.^16^ Cicero, for example, focused on the neurochemical variables involved in more advanced studies of stress, beyond simply inferring tension relief from basic approach‐avoidance behaviors.^17^ Myers (1978, p. 125) celebrated that “the difficult question pertaining to alcohol's multiplicity of effects is now being examined by investigators from different disciplines joining together.” Even McClearn, with his preference for the partial model, still aimed for ever‐more comprehensiveness, breeding strains that drank more, for longer periods, in a wider variety of situations. Selective breeding he argued, offered alcohol research a powerful tool, and “with diligence and application, simulacra developed in this manner may someday grow up to be models” (McClearn, 1979, p. 257).^18^

The CAS was the most active center of research in bringing the various approaches together. In 1962, it moved from Yale to Rutgers, to form a semiautonomous department.^19^ From 1975, it assumed a more formal structure in which the basic research division, headed by David Lester, was “senior” to the other subdivisions.^20^ CAS reports privileged the “animal model of alcoholism” as one of its main objectives, as it “will permit the study of conditions that are not accessible with human subjects. Environmental, genetic and biochemical factors can be controlled and studied and psychological and social influences, such as stress and crowding, investigated.”^21^ Lester and his colleagues, consisting primarily of biochemists, physiologists, and psychologists, continuously developed and applied new approaches, irrespective of their disciplinary origins, such as in the case of SIP and further conditioning procedures to try to realize the criteria.^22^ Lester also worked on selective breeding for sensitivity to alcohol with mice and rats, as well as with conflict and stress approaches, both with his colleague Earl Freed who visited the CAS as an “adjunct appointment.”^23^ This multidisciplinary approach to model building exemplified that of the Center as a whole: “the purposes and needs and methodologies of this or that discipline or this or that profession are not irrelevant to a societal problem center, far from it, but they are *secondary* to the needs of the societal problem field. No discipline and no profession is by itself an adequate basis for resolution of a complex, long‐lasting societal problem.”^24^

Center staff characterized much of the past research into alcohol problems as of a “temporary, ‘one‐shot’ nature,” made in various centers “scattered across the world” and “applied in piecemeal fashion, by, for example, psychiatrists in Boston, a biochemist in Helsinki ….”^25^ The animal model of alcoholism, containing the various elements of heredity, conditioning, and physiological addiction, embodied their vision of the alcohol research field as a whole, and the central integrating role of the CAS within it. It also complemented the other multidisciplinary research approach that followed from the move to Rutgers and directed by Lester—the longitudinal survey—which also sought to identify the various biological‐physical, social, and emotional characteristics associated with drinking problem.^26^

Their vision remained tied to the conceptualization of alcoholism as a disease process, giving the field of alcohol research purpose, unity, and reducing stigma. CAS researchers were acutely aware of the problems of credibility, a proposal to secure further government funding complained of a “particular stigma which has suffused the entire area of any sort of objective study or measured evaluation of programs in the alcohol field.” Individuals of high prestige with only a limited, secondary, or episodic interest in the “resolution of a major national societal problem” received support at the expense of the CAS.^27^ Their pursuit of the animal model related to this issue of credibility. Just as other diseases and medical specialties had their animal models, so to alcoholism, as an equally legitimate physical disease rather than a mere symptom of an underlying illness—its realization in the laboratory would reflect the coming of age of the alcohol research field (cf. Lester, 1982).

Whether a researcher was content with a series of partial models, or determined to combine the various facets into an isomorphic whole, the overarching framework of alcoholism as a progressive disease remained intact. Jellinek's formulation served as the frame through which to integrate the work of various groups of researchers focused on the various components, giving even the most partial of models purpose and legitimacy. The traffic was, therefore, decidedly one‐way: from a conception of alcoholism in the human, translated and represented, in part or in full, in the animal laboratory. In accordance with the criteria established by Lester and Freed, by the late 1970s, SIP was commonly placed among the many varied “forced administration” methods: useful (if time‐consuming and expensive) for examining the effects of excessive drinking on the body, not for uncovering the various psychophysiological motivations that drove the animal to excess (Cicero, 1979, p. 549).

### Redefining the Criteria: The Challenge from Behavioral Pharmacology

The alcohol research field grew rapidly from the mid‐1960s, supported by federal government, as did the mental health field more generally. The CAS became one of a series of national alcohol research centers, supported by the National Institute of Mental Health (NIMH). These centers contributed new approaches and perspectives. The NIMH established its own National Center for the Prevention and Control of Alcoholism in 1966, appointing the research psychiatrist, Jack Mendelson, as its director, and his wife and collaborator, the behaviorist psychologist Nancy Mello, as chief research scientist. Both were becoming leading figures in the emerging field of behavioral pharmacology (or psychopharmacology). For pharmacologists, behavioral methods provided a means of analyzing and quantifying the effects of drugs and predicting their abuse liability; for behaviorist psychologists, the use of pharmacological agents allowed them to develop and promote the importance of the experimental analysis of complex behavioral processes and pathologies.^28^ Addiction research was a central feature of the field; Mello and Mendelson (1971b, p. vi) emphasized how it offered: “definite advantages for the investigation of behavioral and biological interrelationships. In alcoholism, unlike most other major behavioral disorders such as depression and schizophrenia, we can isolate, characterize, and define the agent that is essential for the expression of the disease process. Consequently, it is possible to observe the effects of that agent upon any measurable behavioral, physiological, or biochemical variable as a function of dosage through time.”

As committed as they were to the “medical conceptualization of alcoholism,” they felt that the field was still too reliant on unscientific concepts and anecdotal accounts of the alcoholic during sobriety: “a most unreliable informant” (Mello, 1979, p. 310). In the place of the surveys and interviews used by the CAS, Mello transferred the technologies of operant conditioning from animal laboratory to the ward and clinic. Scientists would now observe, for the very first time, the actual behavior of alcoholics when drinking. Subjects not only received alcohol freely, but also used operant response panels where they had to work for the drug. This allowed for objective measures of intake over a period of weeks, combined with careful observations of behavior and physiological measurements. Mello argued that the research presented “a very different picture of the effects of alcohol on the alcoholic.” They found no evidence of the “impulsive hedonist,” seeking to achieve “a diffuse sense of omnipotence,” no “demonic craving” or “states of oblivion.” Indeed, the idea of “the first drink,” triggering an uninterrupted sequence of compulsive drinking seemed discredited (Mello, 1972, p. 280). They classified the subjects in terms of the “gamma” subspecies of alcoholism, as defined by Jellinek, and yet they controlled their intake, rarely drinking themselves into a stupor, refusing to work for alcohol if the costs were prohibitive, and interrupting bouts with periods of abstinence (Mendelson, 1964). For Mello, the lack of support for the vague, circular, and fatalistic notion of “craving” as the driving mechanism for alcoholic behavior, an approach that was central to the AA program of total abstinence, offered the possibility of a “more rational therapeutic approach to problem drinking” (Mello, 1972, p. 282). Peter Nathan, first directing the Alcohol Study Unit, Boston City Hospital and later, the Alcohol Behavior Research Laboratory at Rutgers, carried out similar studies, identifying how various social and situational factors influenced drinking behavior, and therefore offered possibilities for environmental reinforcement for moderation and remediation (Nathan & Briddell, 1977; Nathan & Lipscomb, 1979; cf. Meyer et al., 1981).

Realizing such an alternative approach required further analysis of the pattern of behavioral and biological variables related to the spontaneous initiation, perpetuation, and cessation of a drinking spree (Mello, 1972, p. 223). This required, in turn, a suitable animal model of alcoholism, one of the central aims of the new NIMH center:

… only an animal preparation will permit study of the developmental sequence and action of possible neurochemical, neurophysiological, and metabolic factors which are concomitants of alcohol addiction. It is postulated that data obtained from analysis of the development of addiction and thereby suggest ways to arrest or reverse the disease process. The eventual goal is to contribute toward a conceptualization of alcoholism that considers both the biological and behavioral determinants of this disorder.^29^

However, in developing this animal preparation, Mello faced the seemingly intractable problem of getting the animal to drink alcohol in pharmacologically significant amounts. First using rats at Harvard, and later rhesus monkeys from the National Institutes of Health primate colony, they applied the techniques of operant conditioning to induce addictive drinking.^30^ They argued that as aversive and rewarding contingencies were at the core of many complex hypotheses of addiction, “a situation in which an animal drinks alcohol as a form of *motivated* behavior in order to avoid pain or to obtain a reward is presumably analogous to the human condition” (Mello & Mendelson, 1971c, p. 314).^31^ However, their use of milk as a reward to induce rats to drink a 10 percent ethanol solution did not result in tolerance or dependence, merely “preference drinking” (Mello & Mendelson, 1965, p. 151). An adapted SIP approach using banana pellets as a reward again failed to induce intoxication or dependency among monkeys, while the pairing of alcohol selection with shock avoidance revealed the determination and ingenuity of the monkeys in learning to simulate a lick response to avoid drinking the alcohol solution (Mello & Mendelson, 1971a, 1971c; Mello, 1973, 1976a). Considering the success of SIP among rhesus monkeys with a nonalcoholic solution (Schuster and Woods, 1966), it seemed that the main objection of the animals to alcohol was its taste. Likewise, with rats, while the animals would drink alcohol, they found it unpalatable at the strength necessary to induce dependency in the limited period that SIP allowed.^32^

With their continued failure using adaptations of SIP, Mello and Mendelson became increasingly interested in an alternative method—that of intravenous administration.^33^ Behavioral pharmacologists at the University of Michigan had studied drug dependence using operant methods—a lever press leading to the injection of an opiate for a physically dependent monkey. By the 1960s, they had progressed to the building of “behavioral chains” whereby a monkey, having learned to lever press for food or the avoidance of shock, would occasionally receive an injection of morphine through a jugular catheter (Thompson & Schuster, 1964). Over time, this developed into a self‐administration pattern for morphine. In 1969, Seevers and his colleagues reported that the monkeys would initiate and then maintain a pattern of self‐administration of morphine, cocaine, and ethanol, having depressed a lever activating an injector out of curiosity (Deneau, Yanagita, & Seevers, 1969). The animals maintained this behavior to the point of inducing motor incoordination, stupor, tremor, vomiting, hallucination, and convulsions—clear signs of physical dependency. The intravenous method by‐passed the issue of palatability and, by reducing the delay between lever press and effect, the animal was better able to associate response with reward. As the animals elected to continue and increase their selection of the drugs, Michigan researchers declared that they had inherent qualities to make them function as reinforcers (Schuster & Thompson, 1969). James Woods reflected on the progress—from the “meagre” demonstration that rats showed a “small” preference for ethanol to “incontrovertible evidence that animals will respond to give themselves intravenous injections of drugs” (1978, p. 595, cf. Winger & Woods, 1973).^34^

By the late 1970s, Mello was declaring that the realization of the animal model of alcohol dependence was one of the singular achievements of behavioral pharmacology and “one of the most significant advances in research in alcoholism” (Mello, 1979, p. 273, cf. Mello, 1985). Through the intravenous method, they had managed to bring together the two partial successes in modeling: animals selecting and self‐administrating ethanol, as seen in earlier preference studies; and in doses sufficient to produce physical dependency, previously only achieved through forced administration. Mello (1976b, p. 347) asserted: “It is now possible to examine the neurophysiological, endocrinological, biochemical and behavioral correlates of the development of alcohol addiction and to study the alcohol withdrawal syndrome in experiments which are neither feasible nor ethical in man.” While they had come close with SIP, one behavioral pharmacologist describing it as on the “brink,” with the intravenous method they now had a “true experimental model of alcoholism.”^35^

As Mello recognized, this approach not only represented a “major methodological advance, but also a necessary conceptual departure from entrenched attitudes that guided research on alcoholism for many years” (Mello, 1985, p. 383). In order to make this model work, Mello first simplified the definition of alcoholism: “Alcohol addiction is defined in terms of the traditional pharmacological criteria of tolerance and dependence” (Mello & Mendelson, 1971c, p. 313). Thus, in its essentials, this definition was consistent with the broad framework of the disease conception of alcoholism as promoted by Jellinek and others at the CAS; and they, in turn, had readily folded into their own criteria Mello's demand the animal select alcohol in its home cage as a measure of dependency. However, Mello's definition also stripped‐out “untestable and circular explanatory concepts such as …‘alcohol craving’ in order to interpret and analyze alcohol self‐administration behavior.” Indeed, for Mello, in accordance with the demands of behaviorism, this was the “special advantage” of the animal model more generally—it required that they leave *a priori* constructs regarding motivations, desires, and cravings at the laboratory door (Mello, 1979, p. 311). Researchers were then “liberated” from “semantic confusions and mentalistic concepts,” freed to study objectively the effects of a stimulus on a behavior (Mello, 1979, p. 309). The necessity of such position seemed justified by the observed behavior of both human and nonhuman primates—in spite of severe withdrawal symptoms, they would interrupt drinking sprees with periods of abstinence, and further, the entire process of alcohol drinking did not appear to induce euphoria or relieve tension, but intensified dysphoria, anxiety, irritability, and aggression. Thus, two of the explanations for the development of alcohol addiction pursued by CAS researchers—pleasure and the avoidance of pain—seemed questionable. For Mello, this raised questions regarding the nature of “punishment” and “reward,” and emphasized the dangers of attributing intrinsic properties to a stimulus event, rather than focusing on “the functional relations between events and behavior” (Mello, 1977, p. 245). While evidence of the reinforcement of supposedly “aversive” events focused behaviorists’ attention onto the importance of the timing of schedule presentation in determining an animal's response, Mello sought to retain a focus on pharmacological effects.^36^ She suggested a process of “stimulus reinforcement”—it was a drug's ability to change a subjective state that gave them abuse potential, rather than their specific individual qualities (Mello, 1979, 1983). This, in turn, further privileged the role of the nonhuman primate as the ideal experimental animal, as “the search for novelty, for varied experience, and for unusual sensation is characteristic of humans and has been observed in higher primates as well” (Mello, 1983, p. 176).

Mello's suggestion of stimulus “state change” reinforcement reflected her commitment to a behavioral pharmacology at the center of the growing field of drug addiction research. Mello and Mendelson left the NIMH in the 1970s to found the Alcohol and Drug Abuse Research Center at Harvard Medical School. Here, they developed comparative research using a variety of stimulants and depressants and focusing on polydrug abuse. The animal model of addiction, by use of the intravenous method, would play a central role in developing a more collaborative approach to addiction research, examining both the differences and resemblances among the effects of various drugs on psychological states, neurobiology, and the central nervous system. The imperative, argued Mello, was to study the reinforcing properties of a variety of drugs, and combine analyses of behavioral effects and biological consequences, so as to “eventually clarify the CNS mechanisms associated with drug reinforcement” (Mello, 1983, p. 187). The fact that the intravenous method contradicted one of the most central criterion established by Lester and Freed, meant that the problem lay with their criteria, not with the model. Mello (1976b, p. 348) argued that the “curious” delay in developing an animal model had resulted from the obsession with the criterion of oral self‐administration:

A pervasive anthropomorphism was probably most responsible for impeding behavioral model development. It was argued that since man drinks alcohol, an adequate animal model of alcoholism must also involve oral consumption and evidence of preference for alcohol in comparison to other fluids. (Mello, 1985, p. 384)

While Lester and Freed had characterized SIP as mere mimicry, this was because they assumed to know already the underlying forces that drove humans to excessive consumption. Moreover, their demand that the animal *drink* like a human, revealed a similar propensity for seeking surface equivalence, or face validity, at the cost of understanding the real functional relationships between events and behavior. It had also inhibited connections between the field of alcohol research and that of drug addiction more generally. Mello remained committed to the conceptualization of alcoholism as a disease, but the only way of achieving an animal model of this process was through relinquishing adherence to restrictive and presumptive criteria that demanded a miniature of the preconceived alcoholic in the animal laboratory. Rather than laboriously piecing together the various partial components of alcoholism in the animal laboratory, they constructed the animal model in its entirety and in its essentials—of choice, tolerance, and dependency—as a means of then interrogating the processes of addiction to understand the various biological and behavioral mechanisms that maintained excessive consumption.

### Retaining the Criteria but Redefining Alcoholism

After a series of short‐term posts in at the Universities of Colorado, Arizona, and Michigan, Falk joined the psychology department at the University of Rutgers in 1969.^37^ It was here, with Peter Nathan's recently established Alcohol Behavior Research Laboratory and the CAS nearby, that Falk began to dedicate himself to the study of SIP in relation to the problems of addictive and excessive behavior. He drew heavily on the research of fellow behaviorists, such as Nathan and Mello, arguing that the clinical evidence of alcoholic drinking contradicted the “official doctrine” that relied upon notions of “craving” and “psychical dependence” to explain the initial “overdrinking” phase identified by Jellinek, which would then lead to physical dependence (Falk & Tang, 1977, pp. 466, 471).

This view presents the alcoholic as akin to a victim we might find in a third‐rate science fiction movie … a motivational zombie who will stop at nothing to satisfy an ever‐increasing hunger for the molecule. (Falk & Tang, 1977, p. 472)

He also drew upon the evidence provided through the intravenous method that revealed “the state of physical dependence on ethanol is analogous to that seen in man” (Falk, Samson, & Tang, 1973, p. 197). Yet, he also noted that it was not sufficient for maintaining addiction as the CAS would define it, as, in spite of the attractiveness of certain drugs as reinforcers and the pain of physical withdrawal, monkeys endured periods of self‐imposed abstinence.

When it came to the criteria for modeling alcoholism in the animal laboratory, Falk's demands were reminiscent of those of Lester and Freed: “It is of prime importance that animals should *drink* ethanol solutions *excessively* and *chronically* if the experimental arrangement is to be considered a model. Drinking, after all, is the concrete behavior which produces alcoholism in humans” (Falk & Tang, 1977, p. 476). To this demand, he also added that the animal freely select alcohol and that physical dependence emerge without the application of extraneous motivational variables such as shock avoidance: “these ‘forcing’ methods cannot be considered as modelling the dynamics of free‐intake overindulgence” (Falk & Tang, 1977, p. 477). As his research progressed, Falk was able to demonstrate physical dependence to the point of dramatic seizures and convulsions by extending the experimental arrangement to a full 24‐hour cycle, involving six feeding periods (Falk, Samson, & Winger, 1972, 1976; Falk, Samson, & Tang, 1973; Samson & Falk, 1975). Falk declared SIP to have finally met the “demanding” standards of “an animal model, possessing the major behavioral and physiological features of the human alcoholic” (Falk, Samson, & Winger, 1972, p. 811).

Falk's position with regard to the valid animal model of alcoholism seems, therefore, somewhat paradoxical, even contradictory. On the one hand, he joined with behaviorists who sought to relieve the field of its “myths” of “motivational destiny,” as demanded by psychological and physical dependence (Falk, 1983). Falk criticized attempts to “produce a miniature version of the definitionally vague ‘alcoholic’” (Falk & Tang, 1988, p. 580). Yet, on the other hand, he adhered to the “official” criteria for modelling established by those at CAS, requiring face validity: that the animal choose to *drink* compulsively and excessively, just like the human, to the point of prolonged intoxication and physical dependence. Indeed, his position on the value of SIP over the intravenous method never wavered, in spite of his close association with Michigan behavioral pharmacologists (Falk, Dews, & Schuster, 1983; Schuster, 2010). He argued that the intravenous route was “more removed from a model of alcoholism” (Falk, Samson, & Winger, 1972, p. 813); and that it “involves a route of administration not chosen by human alcoholics” (Falk & Tang, 1977, p. 474). His position was consistent, however, when we consider what work Falk wished the model to do, or better, what, exactly, he believed he was modeling.

For Falk, the very difficulty in overcoming the animal's aversion to the taste and excessive consumption of alcohol was critical to the model. To do so successfully through SIP privileged the role of environmental factors—the intermittent schedules of reinforcement—in determining behavior.^38^ He further pointed out that when using the intravenous method, the reinforcing qualities of alcohol appeared to be rather weak, the animals often needing to be primed with a cocaine‐based solution; this weakness he had again overcome through SIP. The fact that Falk's animals extinguished their excessive drinking once the experiments had concluded, or when presented with a more attractive solution of dextrose, sucrose, or saccharin, similarly emphasized the importance of environmental factors. The extinction of their excessive behavior was not a problem that needed to be overcome through alternative approaches, but to be understood. Such lack of conformity to the definition of the alcoholic promoted by CAS researchers did not devalue the animal model, but gave it more meaning. With a change in environment came a change in behavior, in spite of their physical dependency on alcohol.

Falk believed that schedule‐induced drinking was analogous to the addictive process in man. The pharmacological properties of the drug were not the crucial factor that determined excess; the schedule was of critical importance. He accepted that his rats were “locked into the situation.” This, however, was part of the model: “Life in many respects is a set of complex intermittent schedules” (Falk, 1981, pp. 328, 331). As commodities of value—food, money, sex—were only intermittently available, alternatives would be sought (Falk, 1983, p. 390). But Falk's rats had few other behavioral alternatives in the experimental situation, just as abusers of alcohol or other drugs had few opportunities or were unable to utilize them effectively:

The prototypical animal experiments using schedule‐induction seem most akin to those conditions characterizing a human subcultural niche (e.g., unemployed ghetto resident) predisposing to alcoholism and other drug abuse. Like the food‐limitation condition of the animal experiments, the static niche or the economically marginal ghetto is also impoverished of reinforcing opportunities that could serve as alternatives to drugs. (Falk & Tang, 1988, p. 580)

Compulsive behavior was not only a problem that befell the ghetto resident, there were numerous ways in which lives were impoverished. The affluent had their share of unfulfilled desires and thwarted ambitions, and Falk was particularly interested in the high levels of drug abuse in professions in which there was uncertainty and intermittency of reinforcement and restricted behavioral outlets, such as among criminals, salesmen, or market speculators. He also interpreted the success of the intravenous method in this light:

Usually there is little in the experimental situation to compete with drug self‐injection, no pre‐existing behavior routines strongly reinforced by agents other than drugs… We make abuse easy for our experimental subjects, thus maximizing the efficacy of the intrinsic properties of a drug to engage behavior, i.e., to function as a durable reinforcer. (Falk, 1983, p. 388)

Yet, unlike the monkey or polydipsic rat, few human beings abused in spite of their exposure to various agents. This was because, for the most part, societies “provide varied sources of reinforcement to their members who also are restrained from spending too much of their time and resources on drugs” (Falk, 1983, p. 388). He drew support from emerging evidence that the high levels of drug‐dependency among soldiers in Vietnam had dissipated rapidly on their return home (Robins, 1974), just as his rats preference of ethanol had dissipated rapidly following their return to their home cages (Falk, 1983).

This helps explain why Falk remained so wedded to the traditional criteria of oral self‐administration leading to physical dependency. Not only did it reveal the power of the “generator schedule” to overcome an animal's intrinsic distaste for excessive alcohol drinking, but even when “addicted” to alcohol, the animal's intake was wholly dependent on the continuation of that schedule, and dissipated once freed from the experimental situation and/or provided with tastier alternative solutions (Samson & Falk, 1974). Falk's model both succeeded and failed: it succeeded in generating compulsive drinking leading to dependence, yet failed to realize the truly alcoholic animal. This was because it was not a model of alcoholism, as defined by CAS researchers. It was a model of excessive behavior more generally, of which alcohol overindulgence was but one example. Alcohol researchers, he argued, had lost track of the purpose of animal modeling. They had first constructed a series of criteria that defined alcoholism as a disease, based largely on “social myths” that endowed terms such as “addiction” and “loss of control,” with “undeserved determining powers” (Falk & Tang, 1988, p. 579). This narrowed their focus, in the language of disease, to the interactions between agent (alcohol) and host (animal). They then tried to build a model to fit this description, forcing a recalcitrant animal into an ill‐fitting cast. While fellow behavioral pharmacologists had achieved much in helping to undermine such long‐held assumptions, the relevance of environmental factors still hinged on the inherent pharmacological value of the drug—contingencies and discriminative stimuli merely helping to maintain drug self‐administration as secondary factors. Through SIP, Falk was able to reverse the order of significance between pharmacology and environment—pharmacological factors were only meaningful in the absence of behavioral alternatives. Alcohol abuse resulted from preexistent excessive behavior, it was a “symptom” not a “disease” (Falk, 1983, p. 386). As Falk argued, the environmental conditions that gave rise to schedule‐induced drinking could also induce a variety of excesses, such as wheel running, aggression, pica, overeating, and, notably, intravenous injections, depending what was available to the animal in the laboratory situation (Hutchinson, Azrin, & Hunt, 1968; Levitsky & Collier, 1968; Flory, 1969; Falk, 1971; King, 1974). Alcohol and drug researchers had not appreciated fully, therefore, the critical role of continuous environmental arrangements.

The emphasis on “generator‐schedule conditions” (Falk & Tang, 1977) had important implications for treatment. As “pharmacological structure does not imply motivational destiny” (Falk, 1983, p. 390), there was reason for optimism. Others agreed, particularly those critical of what they perceived as the medicalization of behavior, resulting in stigmatizing and coercive practices directed toward the “addict” (cf. Szasz, 1974). Through the 1970s, there was growing criticism of the designation of alcoholism as a “disease.” While the purpose of this definition was to aid treatment and research through removing stigma, it assigned blame to the individual, while shifting attention away from broader social and economic inequalities that generated alcohol and drug abuse. It also shifted attention away from alternative forms of treatment beyond that of abstinence, suggested by the clinical studies of alcohol use and a series of surveys that identified alcoholics successfully returning to normal drinking (Armor, Polich, & Stambul, 1976; Pattison et al., 1977). By the 1980s, the psychologist and therapist Stanton Peele was criticizing behaviorists for having betrayed the therapeutic potential of controlled drinking, and for seeking rapprochement with Jellinek's formulation of the disease theory in which “supreme power” was granted to alcohol to “corrupt and control” (1984, p. 1347; cf. Peele 1985, p. 59).^39^ In this context, Falk's work was becoming a popular source of reference (Peele, 1984, p. 1346). Joined by psychologist Bruce Alexander, Peele targeted the reliance on animal experimentation for providing evidence of “the purely physiological genesis of addiction”; and yet, Falk's laboratory experiments helped undermine the idea of an inexorable addiction process (Alexander et al., 1985, p. 73). Alexander constructed “Rat Park”—an airy, spacious, and peaceful space with tins and wood scraps on the floor. When placed in this “psychosocial paradise,” rats would reject morphine, even after sustained periods of forced administration (Alexander, Coambs, & Hadaway, 1978; Alexander et al., 1981; cf. Slater, 2004). Likewise, he argued, drug and alcohol abuse among humans was not the consequence of the intrinsic properties of an intoxicating or stimulating substance, but of a lack of “opportunities for rewarding experiences that characterize life for our species” (Alexander et al., 1985, p. 96).

Falk shared many of the concerns of those critical of the disease approach to alcoholism. He criticized the stigmatization of “underclass” and ethnic groups through their association with the sickness of addiction—the Irish and Native American with alcohol, the Chinese with opium, the African American with heroin and cocaine. Drug abuse was not the cause of deviancy and delinquency, but the consequence of a lack of behavioral opportunities that came with marginalization: “drugs do not have powers to do these things” (Falk, 1983, p. 390). He lent his support to the idea of controlled drinking, but felt that success depended on the person adopting alternative behavioral repertoires more easily practiced and rewarded.^40^ There were excessive behaviors that were productive, such as creative endeavors and workaholic motivations in science, business, and the arts—mirrored by schedule‐induced wheel running in rats, which he even described as “therapy” (Falk & Tang, 1988, p. 583; cf. Fonaroff et al., 1980). For Falk, however, the relevance of SIP extended beyond alcoholism and drug addiction, but to all forms of overindulgence, such as gambling, aggression, sexual activity, eating disorders, and even talkativeness and television viewing: “One of the marks of such behavior is a certain compulsive component that makes the variations in the quality of the agent appear more or less irrelevant” (Fonaroff et al., 1980, p. 102). The attention of scientists and the general public had been “captured by relatively few forms of excessive use,” notably drugs and alcohol; they had, consequently, sought explanations for their abuse in their inherent properties and effects. Falk's use of the animal model was, therefore, far more ambitious than that of the alcohol and drug researchers. It moved beyond the field of alcohol research, represented in an animal model that painstakingly united the various environmental, genetic, and psychophysiological features specific to the human alcoholic; it also moved beyond the broader psychopharmacology of the abuse of numerous drugs, united around the self‐injecting monkey. Through SIP, Falk promoted a new, biologically informed behaviorist psychology that could explain, and potentially control, all forms of persistent and excessive behavior.

## Conclusion

With the development of scientific and medical interest in the problem of alcoholism, animals became critical to its understanding as a disease. Belief in the necessity of an animal preparation for the study of the excessive consumption of alcohol leading to physical dependency united scientific researchers. In establishing animal modeling as central to the field of alcohol research, they undoubtedly succeeded, as Myers (1978) reflected: “No longer is the sight of a rat, hamster, or pig drinking an alcoholic beverage in a laboratory setting greeted by a singular scowl or unrestrained amusement.” However, the issue of success in building an animal model of alcoholism divided opinion. In a letter to Senator Edward Kennedy, CAS Director John Carpenter declared, “unfortunately a satisfactory animal model of alcoholism has not been found.”^41^ Carpenter then referred him to the criteria for an animal model developed by Lester and Freed, which he enclosed. These criteria played an important dual role. On the one hand, the model that they clearly and carefully delineated was an ideal to be worked towards, combining various elements of expertise—pharmacological, genetic, physiological, and psychological.^42^ The model therefore not only embodied the disease of alcoholism, but the very structure of the interdisciplinary and problem‐oriented alcohol research field as established at the CAS. On the other, the criteria served as a means of excluding approaches, such as SIP, which seemed to challenge, even reverse, their understanding of alcoholism as a progressive disease in which an individual began to drink because of the psychologically reinforcing qualities of alcohol, then lost control as alcohol tightened its grip over the physical body and overwhelmed volition. In Falk's model, the animals immediately adopted compulsive drinking behavior, but then, even after months of alcohol abuse, preferred alternative solutions in spite of symptoms of withdrawal. The objection to SIP reveals, therefore, their commitment to a particular definition of alcoholism as a progressive disease, and to a particular kind of modeling in support of that definition, the purpose of which was to replicate and “correspond to essential dimensions and topology of the original” (Lester, 1982, p. 151). In spite of the difficulties in realizing these high standards, and even if they ultimately proved unattainable, it was essential that they be maintained so as to uphold and defend their understanding of alcohol intake as “a strongly reinforcing behavior in some humans, based wholly, it would appear, on the positive central nervous system benefits which alcohol confers. In these humans, drinking alcohol becomes a compulsive need, resistant to change, and a dominant theme of life” (Lester, 1982, p. 153). SIP clearly threatened this understanding, and with it, the very structure and purpose of the alcohol research field.

The very determination to realize a true animal model of alcoholism also generated much critical reflection over the purpose of animal modeling both within and outside the alcohol research field. One scientist, William T. Mckinney (1988, pp. 149, 150), described the continued “search for the elusive, all‐inclusive model” of alcoholism as nothing short of “mysterious,” while another thought it “embarrassing” (Bond, 1984, p. 9). McKinney was one of a number of scientists of psychopathology for whom the value of animal modeling was for understanding common factors underlying a behavioral disorder, rather than attempting to replicate an extremely complex disorder in all its key features, a truly “utopian goal” (Hanin & Usdin, 1977, p. xiii). As he announced, “There is no such thing as a comprehensive animal model of alcoholism, nor will there ever be” (Mckinney, 1988, p. 150). Behavioral pharmacologists were at the forefront of the development of new standards of validity for animal modeling in the 1970s, by which the importance of “face validity” (behavioral similarity) was deemed secondary to “construct validity” (functional equivalence) (cf. Thompson & Unna, 1977; Willner, 1991). The work of Nancy Mello reflected and contributed to such an approach, the animal model becoming less of a replica, and more of a heuristic device. Her objection to SIP was not due to issues of motivation, but due to its failure to generate enduring alcohol preference and physical dependence. By rejecting Lester and Freed's criterion of oral consumption, but retaining the criteria of self‐selection, tolerance, and withdrawal, she declared the model of the intravenous injecting monkey a success, allowing for the analysis of the addictive process at the “behavioral, biochemical and neurophysiological level” (Mello, 1979, p. 273). Once again, the work that this model accomplished was more than scientific, but disciplinary, placing behavioral pharmacology at the center of a new field of addiction research that was no longer divided and restricted according to the focus on a specific drug, such as alcohol.

Falk took a very different approach. The lack of fit between SIP and the phenomenon of alcoholism did not discredit it as a model. He accepted the requirement of face validity regarding the behavior of excessive oral consumption, but reinterpreted the failure of the animals to sustain their heavy drinking outside of the experimental situation as a success. Falk was demanding the scientists follow the behavior of the animals, however unruly and intractable, rather than force their *a priori* distinctions, their preestablished grid, onto their behavior. He emphasized the need to learn from the animals actions, to interrogate, question, and redefine the very problem that they were seeking to model. He sought symmetry between animal and human, reminiscent in many ways of Callon and Latour's demands (Callon, 1986; Latour, 1987). The animals were active in his experimental system, their decisions determining its success; but they were never truly free to choose. Neither were we. Falk stated, “The animals are exposed chronically to ethanol because they are *induced* to drink it *voluntarily*; it is not forced upon them” (Falk & Tang, 1988, p. 577, italics added). There was no contradiction. Choice was always constrained within complex and interactive social and physical environments. The key was to design environments in which the animals made the “right” choices. His laboratory did not fail to produce the alcoholic excesses witnessed in man; on the contrary, the experimental laboratory as whole modeled the world outside. The intermittent feeding machine became an analogue, a “generator schedule,” of the intermittencies that restricted behavior within and outside of the laboratory. In the experimental situation, the rats drank to excess because there were no behavioral alternatives, when in the home cage they did not because there were. Animals did not become alcoholics, because the root of the problem was not physical addiction. Through studying animals, Falk believed that they were able to identify the critical, yet malleable, environmental determinants of addictive behavior, while simultaneously challenging the “social construction of enslavement that works real mischief” (Falk, 1998, p. 97). Thus, once again, we see the prescription for a scientific research field embodied in the model, one that extended beyond the boundaries of alcoholism, and even the broader field of addiction; for Falk, SIP represented nothing less than a new psychology of excessive behavior.

## Footnotes

1 This was a measure of the time it took a rodent to lift its tail from a warming hot plate.

2 As Nichole Nelson (2012) argues in her study of behavioral geneticists engaged in the modeling of anxiety disorders, the development of an animal model is characterized by a continuous process of negotiation, contestation, and framing, as researchers build up a structure of arguments and evidence that link the model and modeled into an “epistemic scaffold” that best fulfills their own methodological and disciplinary interests.

3 For analysis of the different ways in which models teach us both about the world and the processes of modeling, see Morgan (1999).

4 Jellinek (1960, p.1) describes having coined this oft‐used phrase around 1940.

5 Much of Jellinek's understanding of alcoholism had come from a selective study of members of Alcoholics Anonymous (AA), who had answered a self‐reporting questionnaire (the famed meeting of Bill, W., and Dr. Bob, S., having taken place in 1935, and their book, *Alcoholics Anonymous*, published in 1939). Jellinek initially developed a close working relationship with AA, and similarly promoted abstinence as essential to overcoming the disease. Under his direction, the Yale Plan Clinics were the first in the United States to offer outpatient clinical treatment for alcoholism.

6 At the WHO, Jellinek promoted alcoholism as a true addiction, similar to opiate addiction. But he, and the WHO, also recognized it to be “intermediate” in status, between addiction‐producing and habit‐forming, and involving considerable personality factors that meant addiction developed in only a minority of users (Jellinek, 1960, p. 119). Understanding patterns of susceptibility required, of course, considerable scientific research.

7 Sociological work was also central to the CAS, the sociologist Selden Bacon succeeding Jellinek as Director of the Center in 1950. However, the “critical mass” remained in the realm of the biochemical, physiological, and psychopharmacological—see Rutgers Center of Alcohol Studies, Accountability Report, September 1979, CAS Archives, Series 4, Rutgers Center of Alcohol Studies, Box 21. As Jellinek argued, while social, cultural, and economic factors influenced “drinking patterns and the magnitude of the alcohol problems,” psychological and physiological factors had priority as only a minority of individuals “succumb to excessive drinking” (Jellinek, 1960, pp. 19, 20).

8 In the late nineteenth and early twentieth century, scientists in the United States examined the effects of alcohol on a variety of animals—dogs, rabbits, pigs, guinea pigs, monkeys, cats, birds, and rats—as a means of exploring its effects of the human body. This line of research declined rapidly with Prohibition. See, for example, Pauly (1996).

9 Masserman had been trained by Meyer, and Gantt invited by Meyer to direct a new Pavlovian laboratory in psychiatry at Johns Hopkins in 1929—Gerard R. Kelly, “A Last Link to Pavlov: W. Horsley Gantt Reflects,” VA Hospital, Perry Point, W. Horsley Gantt Papers, The Alan Mason Chesney Medical Archives of The Johns Hopkins Medical Institutions, Box 4, Folder 16.

10 See, for example, Adamson and Black (1959), Barry, Wagner, and Miller (1963), Dollard and Miller (1950), Kingham (1958), and Weiss (1958). There remained important differences between those who saw alcohol as allowing an animal to overcome fear and frustration related to a simple approach‐avoidance conflict, and those who remained committed to Masserman's formulation of alcohol disintegrating complex and recently acquired phobias.

11 Epsilon alcoholism was the final species, but was little explored—it involved irregular bouts of intense drinking, but did not seem to involve dependency. Jellinek chose the letters of Greek alphabet to avoid the controversy of selecting terms that were disputed or associated with existing paradigms.

12 Jellinek drew upon Masserman when popularizing the scientific understanding of the tension‐reducing properties of alcohol, even describing his cats as having become “dependent upon alcoholic intoxication; they had become addicted.” “Alcohol, Cats and People,” *The Allied Youth*, Vol. 17, no. 6, 1948, reprinted for circulation by The Yale Center of Alcohol Studies, CAS Archives.

13 One of the most controversial was R. J. Williams’ treatment for alcoholism, along with a range of other diseases, through nutritive supplements, such as the inclusion of glutamine in drinking water (Williams, 1959b). Jellinek was, in contrast, supportive of Mardones’ caution in making links between animal alcohol preference and human alcoholism (Jellinek, 1960, pp. 93–98).

14 Lester, Appendix D‐1, “Self‐maintenance of alcohol intoxication in the rat,” in The Rutgers Center of Alcohol Studies, proposal dated 1966, CAS Archives, Box 5, Folder 17–19.

15 Ibid.

16 Not all were so amenable; Cicero (1979) was very dismissive of assumptions that selective breeding for alcohol preference was a model for alcoholism. Those focused on either environmental or genetic factors would often use the other's techniques as a source of critique—testing preference strains through operant techniques, or vice versa (Mello & Mendelson, 1964; George, 1988). Yet, even so, criticism tended to be restrained and the emphasis was very much on unity among researchers dedicated to the study of alcoholism as a disease process.

17 From the 1960s, studies of stress began to focus on the quality and form of stressor (physical, social, or psychological), its timing and related neurological and physiological processes (Clark & Polish, 1960; Rodgers & Theissen, 1964; Mello & Mendelson, 1966; Freed, 1967; Cicero, Myers, & Black, 1968; Senter & Persenky, 1968; Myers & Cicero, 1969).

18 An example of this layering or folding together of the partial models into a more comprehensive whole is in a symposium dedicated to self‐administration models. In the introduction, H. H. Samson and T.‐K. Li expressed their support for McClearn's position on the value of partial models. They concluded by stating that while they had seen how a variety of important variables were “individually related” to the control of ethanol self‐administration, “An important advance for the future would be the combination of these variables within a single model” (Samson & Li, 1988, p. 572). For a detailed and insightful analysis of the ways in which various behavioral techniques are folded into recent genetic studies using alcohol‐preferring strains of mice, see Nelson (2011).

19 The Center was unpopular among many in Yale, its president included, who objected to its social problem‐oriented nature, independence, and the status of alcoholism as a disease—“it is a symptom of an underlying psychoneuroses. It can, and will be, studied like other neuroses by psychiatrists.” Letter to Norman S. Buck (Provost), June 17, 1959, from Vernon Lippard. The NIMH subsidized the move to Rutgers, and became its main source of funding. See Mark Keller, “History of Move—Yale to Rutgers,” sent to Wendell B. Lipscomb, October 27, 1969, CAS Archives, Box 2, Folder 9–16.

20 The reorganization took place when John A. Carpenter succeeded Bacon as director. Due to the privileged position of basic research, Lester served as “second ranking officer of the Center” and represented the Center on “academic matters.” Center of Alcohol Studies, Annual Report, July 1, 1975–June 30, 1976, CAS Archives, Series 4, Box 20.

21 Rutgers Center of Alcohol Studies, Annual Report, July 1, 1973–June 30, 1974, CAS Archives, Series 4, Box 20.

22 For example, they coupled the animals’ initial contact with alcohol with recovery from sickness (thiamine deficiency), “making it appear to the animal that alcohol possesses medicinal, positively rewarding, values.” Center of Alcohol Studies, Annual Report, July 1, 1975–June 30, 1976, CAS Archives, Series 4, Box 20. See Bass and Lester (1977).

23 Annual Report, Ibid.

24 A Proposal for Support of the Rutgers Center of Alcohol Studies, January 1971, CAS Archives, Box 1, Folder 1–8.

25 Center of Alcohol Studies, Annual Report, July 1, 1972–June 30, 1973, CAS Archives, Series 4, Box 20.

26 The longitudinal survey of alcoholism in New Jersey began in 1973, and from this point, we see survey research taking up most of the Center's basic research resources, followed by animal research. See Lester, The Etiology of Alcoholism: A Longitudinal Study, CAS Archives, Series 4, (Group 3), Box 22a.

27 A Proposal for Support of the Rutgers Center of Alcohol Studies, January 1971, CAS Archives, Box 1, Folder 1–8.

28 Both had come from Harvard, working previously together on alcohol addiction in humans and animals at the Stanley Cobb Laboratories for Psychiatric Research, Massachusetts General Hospital, where Mendelson was director of the biochemistry laboratory—Mendelson to Quarton, Activities of the Biochemistry Laboratory during 1962, January 14, 1963, Erich Lindemann Papers, Countway Library of Medicine, Harvard, Box 1, Folder 26. Harvard was the focal point in the emergence of psychopharmacology with B. F. Skinner, Charles Ferster, and Peter Dews, the Stanley Cobb Professor of Psychiatry and Psychobiology.

29 Annual Report, 1968, Intramural Research Program of the National Center for Prevention and Control of Alcoholism, Center for Alcohol Studies Archives, Rutgers, Series 4, U.S. National Institute of Health, Box 31.

30 While Mello and Mendelson also used rats in their experiments, the choice of monkey reflects their increased availability combined with the close biological relation to man. Clark and Polish (1960) also had early success with avoidance conditioning and alcohol consumption in rhesus monkeys, an approach continued by Mello and Mendelson (1966), and they were also the choice of animal among University of Michigan researchers studying drug dependence (Campbell, 2007).

31 While they were rather vague on this point of comparable environmental contingencies, other behaviorists were more direct. J. D. Keehn argued that the fact that animals “became drunk only when the environment reinforced alcohol drinking” (1979, p. 10), was in itself a suitable model of human drunkenness, which was also socially and environmentally reinforced (cf. Keehn, 1969). Lester and Freed (1973) criticized such an approach, seeing it as another form of forced administration.

32 One of the problems with SIP was that the animals hunger would become satiated and they would no longer work for the food reward, and thus, no longer drink. While, as we shall see, Falk claimed to have induced dependency in his animals, Mello and other researchers had less success.

33 Mello (1972) originally seemed to have mixed feelings regarding the value of the intravenous method as she, and others, felt that the rapid rate of induction would obscure the developmental correlates of addiction. She pursued instead oral self‐administration methods such as SIP.

34 Self‐administration was rarely straightforward, however, and not all the monkeys used by Winger and Woods (1973) were equal in their propensity to self‐administer alcohol intravenously on first exposure. It was often necessary to first train monkeys to self‐administer cocaine under same schedule conditions and then substitute it for alcohol. This variation they considered was reflective of the variation in propensity for addiction among humans.

35 Gail Diane Winger, *Intravenous self‐administration of alcohol by rhesus monkeys*. PhD dissertation, University of Michigan, 1970, p. 55. CAS Library.

36 Of particular importance was the work of Morse and Kelleher (1977), which showed how a supposedly punishing stimulus could become reinforcing monkeys continuously pressing a lever to receive an electric shock they had previously worked so hard to avoid. This focus on the scheduling of events and the subject's experimental history, of which adventitious reinforcement was an example, had significant implications for drug abuse. Various environmental and discriminative stimuli could become part of the reinforcement complex—their presentation leading to repetitive behavior and relapses to drug taking. The classic conditioning model proposed by Abraham Wikler in his work on opiate addiction from the 1940s (cf. Wikler, 1977) was therefore both challenged and advanced by evidence of aversive conditioning—see the papers and discussion involving Mello, Ludwig, and Meyer in Meyer et al. (1981). Morse and Kelleher went even further, however, emphasizing not only that different schedules could influence the effect of a drug, but also that the importance of the schedule of presentation limited the validity of intravenous self‐injection studies as a means of assessing the abuse liability of drugs.

37 At Michigan, Falk was not working with the behavioral pharmacologists, but he did befriend one particularly influential individual and later collaborator, working on self‐administration in monkeys—Charles Schuster (2010). While there is no specific information regarding Falk's appointment at Rutgers, the department was expanding rapidly in the field of experimental psychology in the 1960s, and Falk was one of many experimentalists appointed—see Seymour Rosenberg, *A History of Psychology at Rutgers University*. Retrieved June 4, 2014, from http://psych.rutgers.edu/history‐of‐psychology. The psychology department and CAS were very much separate entities, although there was communication between them, and Falk delivered one the CAS seminars that were designed “to overcome barriers between disciplines”—Center of Alcohol Studies, Annual Report, July 1, 1975–June 30, 1976, CAS Archives, Series 4, Box 20. Peter Nathan would assume directorship of CAS in 1983.

38 Likewise, in his later studies of cocaine, more commonly administered via the oral rather intravenous route in humans, Falk argued that SIP allowed them to overcome the “bitterness” of the solution (Tang & Falk, 1987).

39 Peele was particularly critical of Peter Nathan, accusing him of abandoning controlled drinking as a therapeutic goal due to political and bureaucratic exigencies following his move from the psychology department at Rutgers to the CAS as directorship in 1983, the CAS being abstinence‐oriented (Peele, 1992). Nathan (1992) responded by arguing that it was the discouraging data on the efficacy of controlled drinking therapy over time that had led to his doubts.

40 It is important to note the difference between Falk's position and that of behaviorists, such as Skinner (1972, p. 74), who stated: “We shall not solve the problems of alcoholism and juvenile delinquency by increasing a sense of responsibility. It is the environment which is ‘responsible’ for the objectionable behavior, and it is the environment, not some attribute of the individual, which must be changed.” For Falk, adjunctive behavior was not simply a conditioned reflex or operant behavior determined by the environmental contingencies of the schedule. Falk believed the animals to be active in his experimental system, their behavior “voluntary,” not in the sense of being under the direct control of the will, so much as not being mechanically forced to act. It was possible for an individual to change their behavior, or substitute a response for another, which was then reinforced (Falk, 1984, pp. 106–113). In Falk's therapeutic vision, self‐awareness, self‐control, and individual responsibility were critical.

41 Letter, July 15, 1975, CAS Archives—I am grateful to Judit Ward for this reference.

42 These criteria remain influential in the field—see Ankeny et al. (2014).
